# Long‐Term, Low‐Maintenance, and Impactful: Lessons Learned From Sustaining the NOHARM Pragmatic Clinical Trial

**DOI:** 10.1002/lrh2.70075

**Published:** 2026-08-17

**Authors:** Shritha Gayathri, Amanda Courtright‐Lim, Alexandra Wicker, Jane Hein, Amanda Nelson, Sue Cutshall, Sarah A. Minteer, Andrea Cheville, Jon Tilburt

**Affiliations:** ^1^ University of North Carolina Chapel Hill North Carolina USA; ^2^ Department of Quantitative Health Sciences Mayo Clinic Phoenix Arizona USA; ^3^ Department of Physical Medicine & Rehabilitation Mayo Clinic Rochester Minnesota USA; ^4^ Department of Palliative Medicine Mayo Clinic Rochester Minnesota USA; ^5^ Department of Internal Medicine Mayo Clinic Phoenix Arizona USA

**Keywords:** electronic health records, implementation, non‐pharmacological pain management, pragmatic clinical trials, sustainability

## Abstract

**Introduction:**

Sustaining interventions implemented as part of pragmatic clinical trials requires proactive planning, but how to approach this process remains understudied. The Non‐pharmacological Options in Hospital‐based and Rehabilitation pain Management stepped‐wedge cluster‐randomized pragmatic trial tested an electronic health record‐based educational bundle (known as the Healing After Surgery [HAS] initiative) that encouraged the incorporation of non‐pharmacological pain care in perioperative pain management. The HAS initiative was implemented in multiple surgical practices and hospital sites. One year prior to trial completion, a sustainment committee was established to support the transition of intervention components to clinical ownership and to support posttrial sustainment of the HAS initiative.

**Methods:**

We reviewed meeting minutes from biweekly sustainment committee meetings and conducted debriefing sessions with committee members (informed by the Clinical Sustainability Assessment Tool) to identify sustainment strategies and challenges, which were then organized into meaningful themes.

**Results:**

The following six themes emerged from meeting minutes and debriefings: (1) automation of low‐touch components, (2) reliance on internal champions, (3) intentional handoff communication, (4) institutional attention, (5) relaxed fidelity, and (6) continuous evaluation. Together, they highlight the importance of early, structured planning and adaptable implementation strategies.

**Conclusions:**

Sustainment of the HAS initiative required extensive communication, adaptation, and stakeholder engagement across diverse institutional contexts. Proactive sustainment planning during trial design may help ensure interventions are successfully integrated into routine clinical practice posttrial.

## 1 Introduction

Pragmatic clinical trials evaluate the effectiveness of interventions in routine clinical settings. However, interventions are unlikely to continue once trials end without deliberate sustainment planning [1], even when favorable trial outcomes are observed. Research suggests multiple factors influence the sustainability of interventions, including their adaptability, organizational leadership, and staff resources [2, 3]. However, inconsistent definitions and a lack of theoretically informed assessments of sustainability limit what can be learned from the literature [4, 5, 6, 7, 8]. Well‐known implementation frameworks such as RE‐AIM (Reach, Effectiveness, Adoption, Implementation, Maintenance) and PRISM (Practical, Robust Implementation and Sustainability Model) consider sustainability and maintenance, but even these frameworks may not fully anticipate the obstacles that arise when integrating an intervention into routine clinical practice [9]. These gaps underscore the need to identify strategies to sustain interventions beyond trial settings, but also to evaluate sustainment using theoretically grounded approaches.

Interventions implemented as part of pragmatic clinical trials may have features that facilitate their sustainment (e.g., already having been implemented as part of usual care in routine practice settings to diverse patients), offering insight into how the intervention already functions within existing practice workflows. However, once a clinical trial ends, ownership of the intervention may be transferred from the study team to the practices themselves [1]. Our study team sought to plan for and systematically evaluate this transition using a theory‐informed sustainability assessment approach in the context of the Non‐pharmacological Options in a Postoperative Hospital‐based and Rehabilitation pain Management (NOHARM) stepped‐wedge cluster randomized pragmatic clinical trial. NOHARM was designed to support surgical patients in incorporating non‐pharmacological pain care (NPPC) into their approach to perioperative pain management in conjunction with pharmacological approaches, consistent with the Joint Commission [10] and the Center for Disease Control and Prevention [11] standards.

More specifically, NOHARM tested a bundled electronic health record (EHR)‐based intervention (known as the Healing After Surgery [HAS] initiative) that provided surgical patients with education about NPPC techniques that could be incorporated into their perioperative pain management plan [12]. The HAS initiative was designed to be low touch and was automated so that surgical patients received portal‐based (and later in the trial also mailed) materials and had access to additional web‐based and telephonic resources. Patients could choose their preferred NPPC techniques via a portal‐based guide, which auto‐populated the Epic EHR. Clinical Decision Support (CDS) elements built into the Epic EHR for the NOHARM trial supported care teams in providing patients with education and support for using NPPC.

During the last year of the trial, our team began planning for posttrial sustainment in anticipation of interest in sustaining the HAS initiative among surgical practices. In this paper, we describe our theoretically grounded sustainment efforts and distill lessons that highlight design features and transition strategies future pragmatic trial designers may consider, rather than prescribing a single sustainment model.

## 2 Methods

### 2.1 Setting

The NOHARM trial was conducted across six geographically diverse sites within the Mayo Clinic enterprise (Rochester, Minnesota; Mankato, Minnesota; La Crosse, Wisconsin; Eau Claire, Wisconsin; Phoenix, Arizona; Jacksonville, Florida), encompassing 32 surgical practices. The study began on October 16, 2020, and was completed on May 2, 2024.

### 2.2 Data Collection

Beginning in August 2023 (approximately a year prior to the trial's completion), we developed a work group to identify what might be required for posttrial sustainment. The work group consisted of physician, nursing, physical therapy, and research members of the NOHARM study team. Sustainability meetings were held biweekly starting in August 2023 and are ongoing. During these meetings, sustainment needs and potential resources and processes for addressing these needs were discussed (e.g., transferring the responsibility for various intervention components, identifying strategies for cultivating lasting enthusiasm). Meeting minutes were taken at each sustainability meeting.

### 2.3 Analyses

One co‐author (S.G.) analyzed the meeting minutes for 37 meetings. As trial analyses progressed, we additionally sought to identify key aspects of our posttrial sustainment activities through team debriefings. S.G. held 30‐min discussions with six NOHARM sustainment committee members, guided by the Clinical Sustainability Assessment Tool (CSAT), with particular attention to the domains of “engaged stakeholders,” “workflow integration,” and “organizational context & capacity” [13]. S.G. then synthesized insights from both the meeting minutes and debriefings, presenting them as lessons learned.

## 3 Results

Both meeting minutes and debriefing interviews revealed the following six key themes that are important to consider when moving towards posttrial sustainment: (1) automation of low‐touch components, (2) reliance on internal champions, (3) intentional handoff communication, (4) institutional attention, (5) relaxed fidelity, and (6) continuous evaluation (Table 1). These themes can be broadly grouped into design‐phase considerations (automation of low‐touch components) or transition‐phase considerations (all remaining themes).

**TABLE 1:** Sustainment Themes from the NOHARM Pragmatic Trial.

Automation	Preemptively lessens workflow burden at point of care
Internal champions	Navigate resistance by ensuring NOHARM's core purpose remains at the forefront
Intentional handoff communication	Strengthens intended impact and stakeholder perceptions
Institutional attention	Driver for permanent health system change at the enterprise level
Relaxed fidelity	Leaves room for clinical adaptation that reinforces integration
Continuous evaluation	Enables efficient course correction and posttrial planning

### 3.1 Automation

The design of an intervention significantly influences its long‐term integration and success. Interventions developed with scalability in mind that rely more on automation and less on human resources may have a better chance of sustainment once the trial is over. The HAS initiative, for example, consisted of multiple interconnected components, yet its low‐touch EHR design enabled its automation within a large health system. This characteristic strongly supported the initiative's adoption into new practices by minimizing workflow burden at the point of care. Intervention components like staff training, in contrast, are more difficult to sustain preemptively, as these efforts normally require consistent engagement from the research team. Learning how to automate such processes with limited staff input needs to be a priority during the sustainment period. As ownership of key components shifts to local stakeholders, the intervention's simplicity and scalability become essential for sustainable impact. Pragmatic trials like NOHARM employ bottom‐up implementation strategies, making it especially crucial to design components that are low‐maintenance and minimally intrusive to frontline staff.

### 3.2 Internal Champions

Internal champions may play a pivotal role in facilitating sustainment, as research team priorities inevitably shift from implementing and championing the intervention to data extraction and publication efforts. Posttrial, internal champions can advocate for continuing to deliver the intervention. During the NOHARM trial, the purpose of the HAS initiative resonated with existing advocates of NPPC and those who believed NPPC should be routinely offered alongside pharmacological options for pain care. As one stakeholder put it:

…You need people who have the energy and the enthusiasm to keep speaking about NOHARM. On nursing units, for example, staff who championed NOHARM were the ones who would probably mentor other nurses. This was crucial especially during the pandemic because there were constant staffing turnovers and new nurses. So, if those champions could mentor and be preceptors for the new nurses coming in… I think that's key.

Sustainment, therefore, may be propelled by the determination of individuals who believe in the mission or the broader cultural milieu of the practice environment. However, because internal champions may need to overcome institutional barriers and address gaps in intervention delivery knowledge, internal champions in leadership roles or who have the support of someone in a leadership role may be more effective.

### 3.3 Intentional Handoff Communication

Handoff discussions that balance the benefit conferred to patients if the intervention is sustained, along with any additional asks of stakeholders, can help promote long‐term integration. Stakeholders asked to assume responsibility for intervention components that the study team was previously responsible for may worry about logistical and administrative burdens. However, reinforcing the intervention's benefit to patients can positively shape stakeholder perceptions and enthusiasm. In the context of NOHARM, framing the assigned responsibilities as a mission to educate and empower patients serves as an impactful reminder of the importance of sustaining the intervention.

Additionally, intervention components may need to be adapted so that stakeholders feel capable of sustaining them. For instance, the NOHARM research team worked closely with Mayo Clinic's Health Education & Context Services (HE&CS) to transfer ownership of certain educational components of the intervention. This transition required 6 months of extensive meetings and back‐and‐forth communication, largely focused on reducing the intervention's materials down to a manageable workload. Because of these modifications, the HE&CS team is now able to comfortably sustain these resources.

### 3.4 Institutional Attention

Achieving system‐wide change requires dedicated institutional attention. NOHARM aimed to address a well‐recognized gap in pain care management by providing enterprise‐wide education on NPPC for perioperative care and encouraging patients to take a more active role in their recovery. Leveraging the EHR system, NOHARM tested a systematic approach to integrating NPPC options into routine clinical workflows. For such practices to become permanent, however, leadership support and institutional prioritization are essential. For example, because of our institution's buy‐in, an internal enterprise‐wide committee has agreed to consolidate all HAS information in a single toolkit, ultimately enhancing patient pain care at the physician, nursing, and rehab levels. Without this tangible support, long‐term intervention sustainability may be threatened by a lack of prioritization of the HAS initiative and centralization of resources needed to emphasize its continued importance to existing staff and teach new staff about the initiative.

### 3.5 Relaxed Fidelity

The posttrial period marks an important shift: the need for uniform delivery of the intervention across practices loosens as the intervention is no longer being studied as part of clinical research. This transition creates a new opportunity to modestly tailor the intervention to better fit the distinct workflows of clinical practices without compromising scientific integrity or intervention efficacy.

NOHARM was conducted in real‐world hospital environments and continuously exposed to feedback from various hospital stakeholders. For example, early in the trial, physical therapists at one site reported that a best practice advisory (BPA) within the EHR triggered every time they opened a patient's chart, which interfered with their workflow and led to alert fatigue. However, during the trial, modifications to CDS or intervention materials generally needed to be made universally, and individual practice‐level modifications were more difficult. Any modification was carefully deliberated on to ensure intervention fidelity was maintained and that we were evaluating the same intervention across all participating surgical practices.

During the posttrial period, in contrast, the need for uniformity across all surgical practices has eased. The BPA triggering frequency for physical therapists was subsequently adjusted to align with nursing workflows (once in a 12‐h period rather than at each documentation event), reducing disruption while preserving the intervention's core function of prompting NPPC considerations. This example demonstrates how “relaxed fidelity” in the posttrial period allows for the modification of delivery mechanics rather than intervention content, which may enhance workflow compatibility and facilitate sustainment without undermining the intervention's intended effect.

### 3.6 Continuous Evaluation

While adherence to trial fidelity may not allow for immediate improvements to an intervention, tracking and monitoring feedback from stakeholders can support intervention tailoring and adaptation during the posttrial period. Usage data on different components can also help distinguish core mechanisms from supportive elements when making sustainment decisions. For instance, due to favorable levels of engagement with the HAS website during and after the trial, this intervention component has been prioritized for sustainment. In contrast, patient‐facing Zoom calls, which were offered as optional support resources to reinforce patients' use of NPPC, were not sustained. These calls were attended by a low subset of patients (< 1%), suggesting that they were unlikely to account for the overall intervention effects observed during the trial. Given that the intervention's primary mechanism operated through EHR‐enabled clinician education and patient‐facing digital resources, retiring the Zoom calls was viewed as unlikely to compromise efficacy. Such insights allow researchers to “let go” of low‐uptake, supportive components while preserving the intervention's core elements.

Together, these themes supported posttrial sustainment of the HAS initiative. Following the conclusion of the trial, the intervention continued across all 32 original participating surgical practices. Moreover, the intervention has since been adopted by additional practices within the health system, providing pragmatic evidence of sustained implementation beyond the trial period.

## 4 Limitations

These lessons should be interpreted with due caution. Sustainment activities occurred within the context of a large, well‐resourced health system, limiting transferability to other, less centralized organizations. The HAS initiative was also implemented over a relatively short time frame, which may limit the extent to which our findings capture sustainability challenges associated with longer‐duration interventions. Interventions requiring prolonged or ongoing patient engagement may face additional sustainment barriers, such as the need for different reinforcement strategies, which were not a focus of the present study. Additionally, sustained intervention engagement posttrial may be influenced by the loss‐of‐trial‐related contact (e.g., informed consent processes) and the shift from research participation to routine clinical recommendation. While this theme did not emerge in our committee discussions, it may be particularly salient for longer‐term interventions where trial‐related accountability and researcher contact play a larger role. Sustainment efforts within this context may require compensatory strategies, such as enhanced clinical ownership or alternative engagement mechanisms, to replicate elements of support present during trial participation. Furthermore, data were derived from internal stakeholders closely involved with the intervention, offering valuable insider interpretations but also raising the possibility of bias. Finally, the NOHARM trial was launched during the COVID‐19 pandemic, and some lessons—particularly the reliance on champions during times of workforce turnover—may reflect pandemic‐specific circumstances. Nevertheless, many of the overarching lessons are expected to remain pertinent for maintaining practical trial interventions after the pandemic. Future work should examine whether these lessons hold in more typical healthcare environments, as well as in settings that differ in scale, infrastructure, and available resources. Future work should also examine whether these lessons are applicable to longer‐term interventions.

## 5 Conclusion

Implementing NOHARM required collaboration among numerous stakeholders across multiple local institutional environments, with resilient communication and persistent engagement proving especially critical during the sustainment period.

Six thematic domains capture our lessons learned for sustaining pragmatic trial interventions posttrial, the majority of which relate to the transition from active trial management to routine clinical ownership. Our findings highlight the importance of transition‐phase strategies such as identifying and supporting internal champions, facilitating intentional handoffs, and embracing adaptation alongside ongoing monitoring. At the same time, certain design features, particularly the automation of low‐touch components, can ease this transition when sustainment becomes relevant. Importantly, these findings are not intended to suggest that all pragmatic trials must invest in identical, resource‐intensive infrastructures such as formal committees. Rather, our experience demonstrates that a combination of thoughtful intervention design and pragmatic, context‐sensitive transition strategies can substantially reduce the burden of posttrial handoff and support sustainment in routine practice.

Clinical research is driven by meeting deadlines and submitting deliverables, moving at a pace that does not always match robust posttrial intervention sustainment efforts. Yet, our findings highlight a need for dedicated reflection and resilient communication to successfully actualize the transition of pragmatic trial research into clinical practice. Further research attention should focus on proactively assessing and planning for postpragmatic trial sustainment to enhance its impact.

## Funding

This work was supported by the National Institutes of Health (UG3AG067593, UH3AG067593, U24AT010961) and PRISM Resource Coordinating Center.

## Conflicts of Interest

The authors declare no conflicts of interest.

## Data Availability

Data sharing not applicable to this article as no datasets were generated or analysed during the current study.
